# Hepatobiliary Manifestations of Sickle Cell Anemia

**DOI:** 10.4021/gr2010.01.1332

**Published:** 2010-01-20

**Authors:** Hussain Issa, Ahmed H. Al-Salem

**Affiliations:** a Department of Internal Medicine, Division of Gastroenterology, King Fahad Specialist Hospital, Dammam, Saudi Arabia; b Department of Pediatric Surgery, Maternity and Children Hospital, Dammam, Saudi Arabia

**Keywords:** Sickle cell anemia, Hepatobiliary complications

## Abstract

Sickle cell anemia is one of the common hemoglobinopathies around the world. It results from a single change of one amino acid valine instead of glutamic acid in the hemoglobin beta change. This change leads to polymerization of the hemoglobin when the oxygen saturation is lowered, resulting in deformity of the red blood cells and microvascular occlusion. Sickle cell anemia can affect any part of the body and one of the main organs to be affected is the hepatobiliary system either directly from the sicklening process or indirectly as a result of chronic hemolysis and multiple blood transfusions. This manifests in several clinical conditions which poses diagnostic and therapeutic dilemmas to the treating physicians. These hepatobiliary manifestations will be outlined in this review.

## Introduction

Sickle cells were first recognized in 1904 by the Chicago cardiologist and professor of medicine James B. Herrick (1861-1954) [1]. His intern Ernest Edward Irons (1877-1959), while examining a peripheral blood smear from Walter Clement Noel, a first-year dental student from Grenada who was admitted to the Chicago Presbyterian Hospital suffering from anemia, found “peculiar elongated and sickle-shaped” red blood cells. Vernon Mason in 1922 named it “sickle-cell anemia” [2]. In 1949, Linus Pauling and his colleagues were the first to demonstrate that sickle-cell anemia (SCA) occurs as a result of an abnormality in the hemoglobin molecule [3]. It is now well established that SCA results from a single change of one amino acid, valine instead of glutamic acid at the sixth position among the 146 amino acids of the hemoglobin beta chain [4, 5]. A single change of one amino acid can lead to so much morbidity and mortality. This change leads to polymerization of the hemoglobin when the oxygen saturation is lowered, resulting in deformity of the red blood cells and microvascular occlusion. This as well as its subsequent effects including cellular dehydration, inflammatory response and reperfusion injury which are important pathophysiological mechanisms leads to the different manifestations of SCA. In Saudi Arabia, SCA is common and one of the most affected regions is The Eastern Province where the frequency of sickle cell trait and sickle cell anemia can reach as high as 25% and 2% respectively in some areas [6, 7].

SCA can affect any part of the body and poses diagnostic and therapeutic dilemmas to the treating physicians. The hepatobiliary system is one of the common organs to be affected either directly from the sicklening process or indirectly as a result of chronic hemolysis and multiple blood transfusions. This can manifest in several clinical conditions which will be outlined in this review. The role of surgery in relation to the different hepatobiliary complications will be discussed also.

## Cholelithiasis

**Figure 1:**
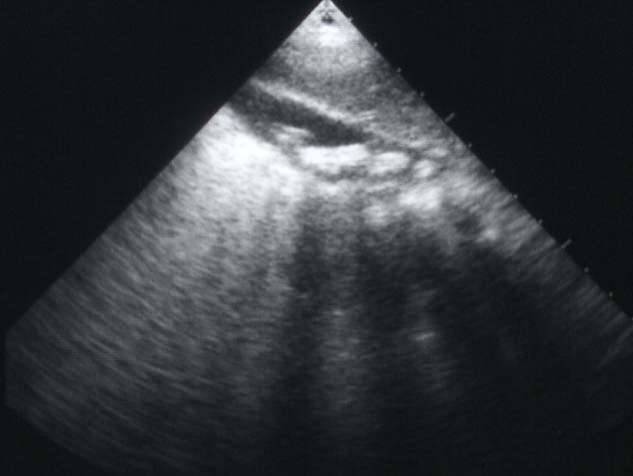
Abdominal ultrasound showing multiple gallstones.

Cholelithiasis is one of the common complications of SCA (Fig. 1). These are usually pigment stones that result from chronic hemolysis leading to increased bilirubin production. The frequency of this however is variable depending on the age of the patients and the diagnostic method used. A frequency ranging from 5%-55% has been reported but an overall 70% of patients with SCA will develop gallstones at one stage of their life [8-12]. With better understanding of SCA, improved medical and surgical management of these patients and the development of new drugs such as hydroxyurea have contributed to a better survival and increased life expectancy of these patients. This will lead to an increased frequency of cholelithiasis in these patients as the frequency of gallstones in patients with SCA increases with age. In a previous study on 305 children with SCA, we found an overall 19.7% frequency of gallstones. This increased from 8.7% in those less than 10 years of age to 36% in those 15-18 years of age [8]. The treatment of cholelithiasis in patients with SCA is cholecystectomy. In the era of minimal invasive surgery, laparoscopic cholecystectomy is now the procedure of choice. This is specially so for patients with SCA where it was shown to be feasible, beneficial and safe [13-15]. The fact that there is no large abdominal wound, laparoscopic cholecystectomy should be advantageous to patients with SCA. Not only the hospital stay is short and cosmetically it is better but the associated postoperative pain is much less. This makes their early mobilization much easier and will not interfere with their breathing which may prove beneficial in reducing the incidence of postoperative acute chest syndrome.

**Figure 2:**
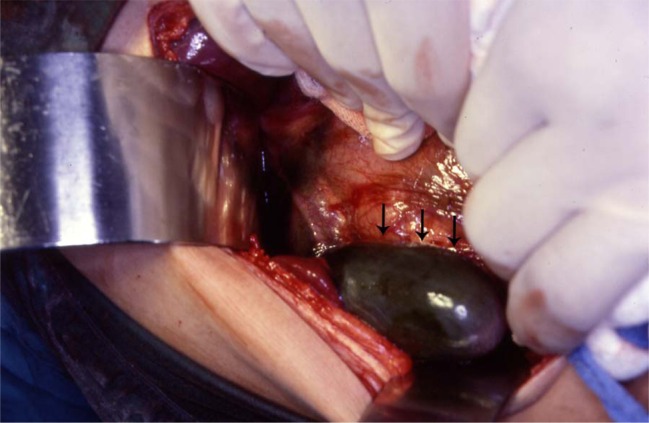
Intraoperative photograph showing acute gangrenous cholecystitis in a patient with SCA.

The treatment of asymptomatic gallstones in patients with SCA is still controversial. In the past and because of a reported perioperative mortality rate as high as 10% and a postoperative complication rate up to 50% surgery in patients with SCA was not advocated except in symptomatic patients [16-18]. This however is not the case nowadays as with better understanding of SCA and improved perioperative care, surgery is safer. We like others advocate cholecystectomy for asymptomatic gallstones in patients with SCA [19]. The life expectancy of patients with SCA is known to be shorter than the general population and they usually die of SCA related complications but as a result of better understanding of SCA and its complications, the use of hydroxyurea as well as improved medical and surgical care, SCA patients are now living longer [20-22]. This makes them liable to develop gallstones related complications including biliary colic, acute cholecystitis, choledocholithiasis, obstructive jaundice, ascending cholangitis and pancreatitis [23]. Operating on these patients on an emergency basis, not fully prepared is definitely associated with a high morbidity and mortality (Fig. 2). It is much better to operate on these patients electively when they are well prepared [24]. Add to this the fact that cholecystectomy should simplify the future management of abdominal crisis as the possibility of acute cholecystitis is eliminated. It has been estimated that about 70% of patients with SCA will develop gallstones at one stage of their life [12]. This is a high frequency and a question that is being raised nowadays is whether this justifies incidental cholecystectomy for SCA patients who are having abdominal surgery electively for some other reason such as splenectomy, hiatal hernia repair? This is still controversial and most surgeons are not supportive of this.

## Choledocholithiasis

**Figure 3:**
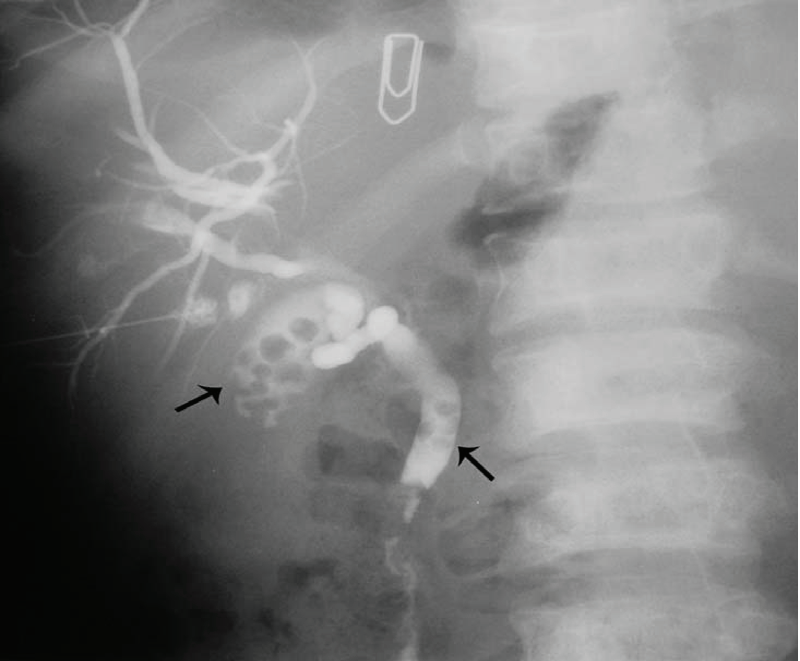
Percutaneous cholangiogram showing gallstones and common bile duct stones.

**Figure 4:**
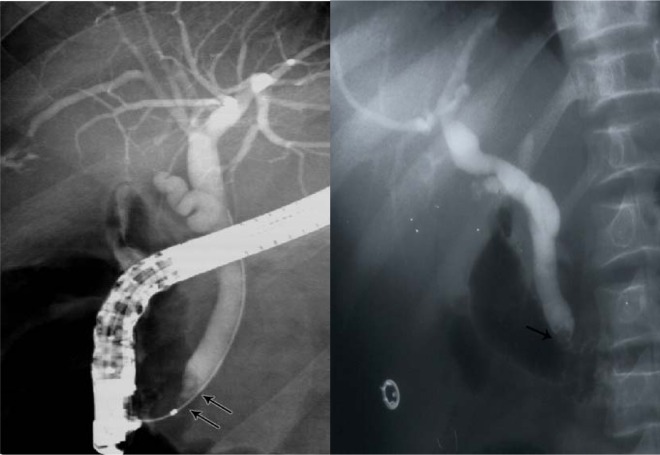
ERCP showing bile duct stones pre and post laparoscopic cholecystectomy.

Another relatively common complication of SCA is the development of choledocholithiasis which is usually secondary to cholelithiasis but there are also primary choledocholithiasis [25] (Fig. 3). In the general population with cholelithiasis, the incidence of common bile duct stones has been reported as 10%-15% [23]. In patients with SCA, the frequency of common bile duct stones is 18%-30% [23, 25, 26]. We found a 30% incidence of common bile duct stones in children with SCA undergoing cholecystectomy [26]. As a result of this high incidence of choledocholithiasis, routine intraoperative cholangiogram was advocated for those undergoing cholecystectomy [27]. In the era of laparoscopy and endoscopic retrograde cholangiopancreatography (ERCP), the question is whether laproscopic cholangiography with or without common bile duct exploration is necessary in patients with SCA [28]. We do not perform laparoscopic cholangiography as this will increase the operative time, and makes it difficult to decide when CBD stones are diagnosed whether to convert the operation to open cholecystectomy or wait and do a post laparoscopic cholecystectomy ERCP. Add to this a 25% false positive rate that may lead to unnecessary CBD exploration or conversion to open cholecystectomy [28]. We found ERCP valuable both as a diagnostic and therapeutic procedure for SCA patients with choledocholithiasis both pre and post laparoscopic cholecystectomy (Fig. 4). This sequential approach of endoscopic sphincterotomy and stone extraction followed by laparoscopic cholecystectomy is safe and effective approach for the management of SCA patients with cholelithiasis and choledocholithiasis [29, 30]. Since we adopted this approach, none of our patients required common bile duct exploration.

## Biliary sludge

**Figure 5:**
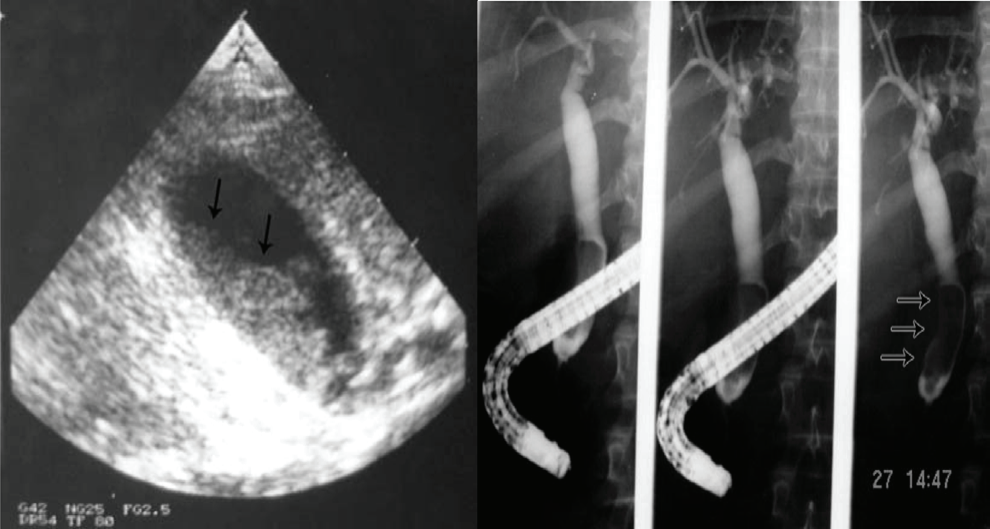
Biliary sludge in the gallbladder and bile ducts.

This is one of the common and interesting complications of SCA. Biliary sludge which is a sequel of chronic hemolysis, is a mixture of calcium bilirubinate and cholesterol crystals within viscous bile that contains a high concentration of mucus and proteins. It can be seen in the gallbladder or bile ducts (Fig. 5). On ultrasound, it characteristically produces a low-amplitude echo pattern, intraluminal layers in the dependent part of the gallbladder. In a previous study on 305 children with SCA, 50 (16.4%) of them had biliary sludge [8]. The question that is always asked is what is the significance of biliary sludge [31]? In a prospective study and follow-up of the 50 patients with biliary sludge, the majority of them (65.7%) developed gallstones [32]. These patients with biliary sludge should be followed-up and those who develop gallstones, they undergo elective laparoscopic cholecystectomy. We also had patients who were symptomatic because of biliary sludge and these patients should be offered laparoscopic cholecystectomy to relief them from their symptoms. A question that still needs an answer is since the majority of patients with biliary sludge will develop gallstones, is it advisable to offer these patients whether symptomatic or not elective laparoscopic cholecystectomy? There are those who advocate conservative treatment and follow-up of patients with biliary sludge and offer them laparoscopic cholecystectomy when they develop gallstones while others and based on the high rate of development of cholelithiasis in these patients advocate elective laparoscopic cholecystectomy [32, 33]. This will obviate the subsequent risk of developing biliary sludge and Cholelithiasis related complications.

## Sickle cell cholangiopathy

**Figure 6:**
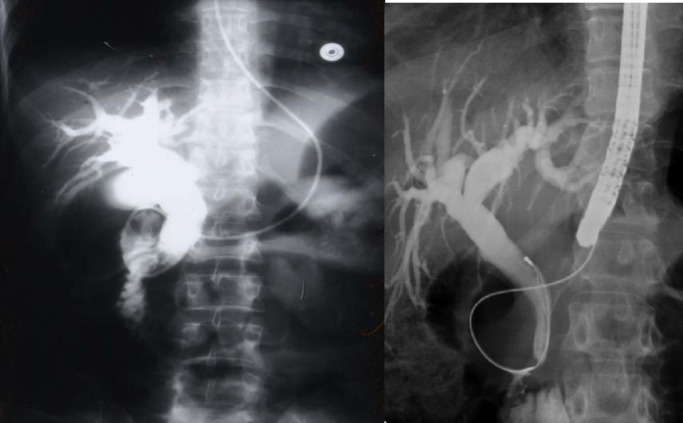
ERCP showing dilated bile ducts without an obstructive cause (Sickle cell cholangiopathy).

This is an interesting but still not well established complication of SCA [34, 35]. It is seen in patients who present with cholestatic jaundice and on ultrasound and ERCP are found to have bile duct dilatation without an obstructive cause (Fig. 6). We have evaluated 224 SCA patients who had ERCP as part of their work up for cholestatic jaundice and 50 (24.6%) of them were found to have bile duct dilatation without an obstructive cause [36]. The reason for this dilation is not exactly known and none of them had previously cholelithiasis or choledocholithiasis. We feel this is a form of cholangiopathy that is a consequence of sickling in the end arteries of the biliary arterial tree leading to hypoxia and dilatation. The bile ducts are supplied via the hepatic arteries and ischemic bile duct injury may occur when these vessels are injured or occluded. This ultimately will result in ischemia of the bile ducts and the effect depends on the extent and velocity of the occlusive process. In patients with SCA, we feel that the occlusion which is usually not complete is of the peribiliary vascular plexus and is a result of sickling within these vascular channels. This ultimately will lead to hypoxia of the bile ducts leading to their dilatation rather than ischemia and stricture formation. This is SCA cholangiopathy and the extent of this is also variable. We found patients with bile ducts dilatation limited to the common bile duct but there are also those who had dilatation involving both extra and intrahepatic bile ducts. Documenting this is of great importance as these patients need to be followed up regularly for the possibility of developing bile duct stones. Taking in consideration the high frequency of biliary sludge and the possibility of bile duct stones formation in these patients, endoscopic sphincterotomy may be beneficial as this may obviate the future development of bile duct stones [36, 37]. This as well as the value of endoscopic sphincterotomy in this group of patients needs however to be evaluated further.

## Pancreatitis

Pancreatitis is not a common complication of SCA. Commonly, it is seen in patients with cholelithiasis as a result of common bile duct obstruction but there are reports of pancreatitis in patients with SCA without cholelithiasis. In these patients, pancreatitis may be due to microvascular occlusion and ischemic injury to the pancreas [38]. Acute pancreatitis should be considered in the differential diagnosis of abdominal pain in patients with SCA. There are however reports of pancreatitis in SCA following ERCP [37]. These are usually mild and transient and procedure related complications. In a review of 49 pancreatitis cases seen in children, eight of them had SCA and five underwent cholecystectomy with no further episodes of pancreatitis [39]. Two other case reports described pancreatitis in a 51-year old SCA patient who developed pancreatic pseudocyst and another 3 year old girl who developed pancreatitis with no evidence of biliary tract pathology [40, 41]. The treatment of pancreatitis is usually conservative with intravenous hydration, antibiotics and analgesia. Laparoscopic cholecystectomy is advocated in those with gallstones related pancreatitis. This should be done electively once their condition becomes stable to avoid further attacks.

## Sickle cell hepatopathy

One of the common manifestations of SCA is jaundice which can be caused by a variety of hepatobiliary diseases including cholestatic jaundice [42-46]. There are however certain causes of cholestatic jaundice that are SCA related. One of these is intrahepatic sickling of RBC [44, 45]. This can lead to cholestasis and a clinical picture that may resemble extrahepatic bile duct obstruction which causes diagnostic and therapeutic dilemmas.

Intrahepatic sickling produces a constellation of signs and symptoms and although they are divided into distinct clinical syndromes there is however overlap between them and the overall effect is called sickle cell hepatopathy [45]. Early recognition of these syndromes, diagnosis, and prompt treatment are important to achieve a favorable outcome. The aim should be early diagnosis and treatment to prevent irreversible liver injury and every mean possible including the laboratory findings, radiological investigations, and liver biopsy should be utilized to establish the diagnosis.

## Acute sickle cell hepatic crisis

The exact etiology of acute hepatic crisis is not known. It is thought to result from stagnation of sickled red blood cells within the liver sinusoids [42, 46]. This will result in a decrease in circulation through hepatic sinusoids. The clinical presentation of acute liver cell hepatic crisis is variable depending on the severity but the majority present with right upper quadrant abdominal pain, low grade fever, and vomiting. This is usually associated with leukocytosis and mild to moderated elevations of the liver enzymes and bilirubin levels. The increase in bilirubin is that of the conjugated part. The treatment is conservative and supportive as it is self limiting and it usually resolves spontaneously. In severe cases, simple or partial exchange blood transfusion may be necessary. An extremely rare complication of acute severe hepatic crisis that must be kept in mind is hepatic infarction [46]. This is usually seen as a characteristic wedge-shaped, peripherally located hypodense lesion on CT scan [47].

## Hepatic sequestration crisis

Sequestration of red blood cells is a common complication of SCA. This is commonly seen in the lungs and spleen [45]. Acute splenic sequestration crisis is considered the second most common cause of death after infection during the first 5 years of life in children with SCA [48]. Hepatic sequestration crisis is not common and it is caused by obstruction of the blood flow from the liver sinusoids by the sickled red blood cells leading to compression of the bile ducts [42, 46]. This will lead to pooling of blood within the liver leading to acute hepatic enlargement. It is associated with a rapid drop in hemoglobin and hematocrit levels and an increase in the reticulocyte count and bilirubin level [49, 50]. This differentiates it from acute hepatic crisis. The liver enzymes are only mildly elevated and the hyperbilirubinemia is that of the conjugated fraction. Once diagnosed and treated with hydration and blood transfusion, it behaves like splenic sequestration with regression of the hepatic size and increase in hemoglobin level. Recurrence is common and a chronic hepatic sequestration form was also described [51].

## Acute sickle cell intrahepatic cholestasis

Acute sickle cell intrahepatic cholestasis is a rare but a serious and sometimes fatal complication of SCA [46, 51-53]. It is a distinct clinical entity but some consider it a severe form of sickle cell hepatic crisis. It results from sever obstruction of the liver sinusoids leading to stasis, hypoxia and intracanlicular cholestasis secondary to ballooning of the hepatocytes. Clinically, it resembles acute hepatic crisis but the main differentiating points are the associated extreme hyperbilirubinemia, coagulopathy and acute hepatic failure. Early recognition of this potentially fatal condition is crucial as ultimately it will lead to liver failure, encephalopathy and renal impairment. Early diagnosis, intensive supportive care including exchange blood transfusion, fresh frozen plasma, platelet transfusion and plasmapheresis may reverse the process of intrahepatic sickling and cholestasis resulting in a favorable outcome [51].

A variant of chronic intrahepatic cholestasis was also described. This is a mild benign condition characterized by prolonged hyperbilirubinemia in the absence of right upper guardant abdominal pain and evidence of hemolysis [43, 46, 54]. Once it is recognized, it can be successfully treated with a regular exchange blood transfusion program with or without hydroxyurea. A similar condition of benign hyperbilirubinemia was also described in children with SCA [54].

## Hepatitis B and C

**Figure 7:**
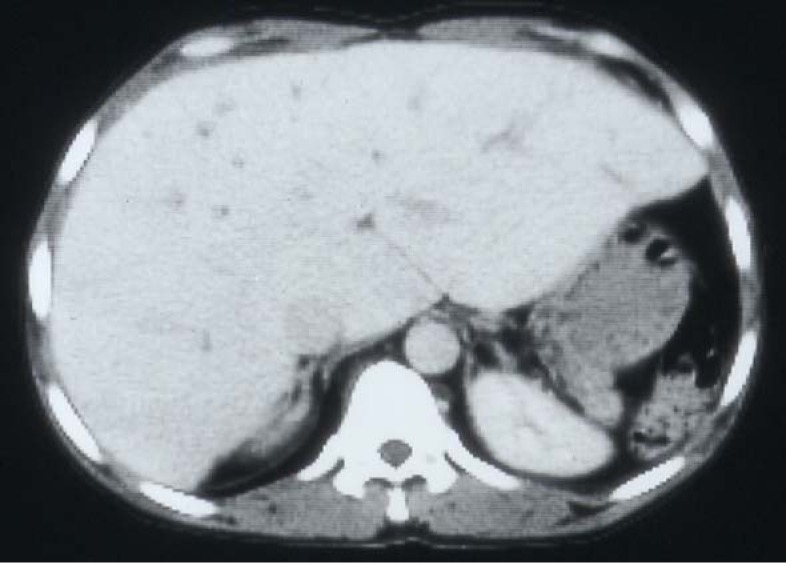
CT-scan of the abdomen showing the liver with iron overload in a patient with SCA and multiple blood transfusions.

SCA is a chronic illness characterized by periods of remissions and exacerbations which are called crisis. One of these is hemolytic crisis. Although there is a chronic hemolysis in patients with SCA, they can also have an acute hemolytic crisis. This will lead to acute anemia which will require blood transfusion. Add to this the frequent need of blood transfusions for other complications of SCA such as splenic sequestration crisis, hepatic sequestration, acute chest syndrome, priapism and central nervous system crisis. Add to this the need for blood transfusion as part of their preoperative preparation for major surgery. This makes them liable to develop blood transfusion related complications including iron overload and hepatitis B and C (Fig. 7). Hepatic iron overload is a serious complication of chronic transfusion therapy in patients with SCA. The degree of liver iron overload is clearly associated with the number of previous blood transfusions [55, 56]. This will lead to liver fibrosis and, eventually, liver cirrhosis.

There is a definite increase in the prevalence of hepatitis B and C infections in patients with SCA. Johnson et al. found evidence for hepatitis B infection in 19% of patients with SCA [57]. In another study, hepatitis B core antigen was positive in 14% of patients with SCA [58].The prevalence of chronic hepatic B in patients with SCA is reported to be low, less than 3.3%, but in parts of the world where hepatitis B is more prevalent, the rate is higher [46]. One important contributing factor for this is the availability of vaccination against hepatitis B. Hence early vaccination against HBV would probably be the only effective way of controlling HBV infection in these patients. This is in contrast to hepatitis C where so far no vaccination against it is available. Preventive measures such as blood screening for anti-HCV before transfusion and stringent infection control measures are crucial steps to be implemented for the control of spread of HCV among these patients. Bahakim et al showed that HCV is endemic in the Saudi population with an overall frequency of 5.3% in healthy Saudi adults which is at least 5 times higher than what has been reported from Western Europe and the United States [59]. The frequency of hepatitis C is much higher in those with SCA but this is variable. Hassan et al in a total of 99 patients found antibodies to HCV in 10 (10.10%) [60]. Torres et al tested 291 patients with SCA for the presence of anti-HCV antibodies and found a 14.1% prevalence [61]. In patients with SCA, the prevalence of HCV antibody is directly related to the number of blood transfusions. Richard et al found that patients who received more than 10 blood transfusions had a 26.4% prevalence of anti-HCV antibodies, in comparison to 10.7% in those who received less than 10 blood transfusions [58]. Devault et al in 121 consecutive patients with SCA detected anti-HCV antibodies in 25 (20.7%) of them [62]. The prevalence of anti-HCV antibodies was 30.3% in those who received more than 10 units of blood products, in comparisons to 8.6% in those who received less than 10 units [62].

Patients with chronic hepatitis C if left untreated are susceptible for liver damage, liver cirrhosis, hepatocellular carcinoma and live failure. Add to this the fact that the combination of iron overload and chronic hepatitis C can lead to more rapidly progressive liver disease. In the past there was no well proven treatment for chronic hepatitis C, but the recent progress has made treatment and cure of chronic hepatitis C possible. Patients with SCA and chronic hepatitis C for many years were considered to be unsuitable for such treatment. One reason for this is that ribavirin may induce hemolysis which can further aggravate their already existing anemia. This is not the case nowadays and patients with SCA and chronic hepatitis C can be treated with pegylated interferon and ribavirin [63-66]. The treatment is safe and effective and the response rate is comparable to those patients without SCA.
